# Periarticular screws: what’s in and what’s out of the joint?

**DOI:** 10.1186/s12891-021-04928-9

**Published:** 2022-01-06

**Authors:** Michael S. Sridhar, Michael D. Hunter, Michael J. Colello

**Affiliations:** Prisma Health-Upstate Department of Orthopaedic Surgery, University of South Carolina School of Medicine Greenville, 701 Grove Road, 2nd Floor Support Tower, Greenville, SC 29605 USA

**Keywords:** Periarticular, Screw, Fluoroscopy, Intraoperative, Hip, Humerus

## Abstract

Periarticular hardware placement can be challenging and a source of angst for orthopaedic surgeons due to fear of penetrating the articular surface and causing undue harm to the joint. In recent years, many surgeons have turned to computed tomography (CT) and other intraoperative or postoperative modalities to determine whether hardware is truly extraarticular in areas of complex anatomy. Yet, these adjuncts are expensive, time consuming, and often unnecessary given the advancement in understanding of intraoperative fluoroscopy. We present a review article with the goal of empowering surgeons to leave the operating room, with fluoroscopy alone, assured that all hardware is beneath the articular surface that is being worked on. By understanding a simple concept, surgeons can extrapolate the information in this article to any joint and bony surface in the body. While targeted at both residents and surgeons who may not have completed a trauma fellowship, this review can benefit all orthopaedic surgeons alike.

## Background

Placing periarticular hardware is a daily practice among fracture surgeons. One of the challenges with these cases is the radiographic confirmation of safe, extraarticular fixation. Multiple articles have been published on surgical techniques to avoid intraarticular screw violation, but there is a paucity of review literature to guide trainees regarding ways to use fluoroscopy alone to ensure extraarticular placement during surgery. Because of the morbidity caused by inadvertent intraarticular screws including pain and mechanical blocks to motion, need for reoperation, chondral damage, and avascular necrosis, avoiding this complication is of the utmost importance [1]. Chengla et al. discussed proper fluoroscopic evaluation for placement of acetabular screws to minimize the risk of articular penetration [2]. A similar study was conducted by Norris et al. which confirmed extraarticular screw placement in all 32 acetabular fractures using fluoroscopy alone [3]. Additionally, several other historical and more modern techniques have been described to further evaluate for articular penetration of the hip including auscultation [4], arthroscopy [5], and postoperative CT scanning [6]. Postoperative plain films are also used at some centers. However, many of these methods require increased operative time, cost, and with CT scanning further potential morbidity is introduced from radiation exposure. Another disadvantage of these techniques is that some do not allow the surgeon to make immediate changes in the operating room, thus requiring a subsequent procedure if intraarticular hardware is detected postoperatively. This can be stressful for the surgeon and patient after already enduring a challenging periarticular fracture fixation case. In this article, we present our practical fluoroscopic methodology to avoid these issues and allow for confident placement of subarticular screws.

## Rationale

**Fig. 1:**
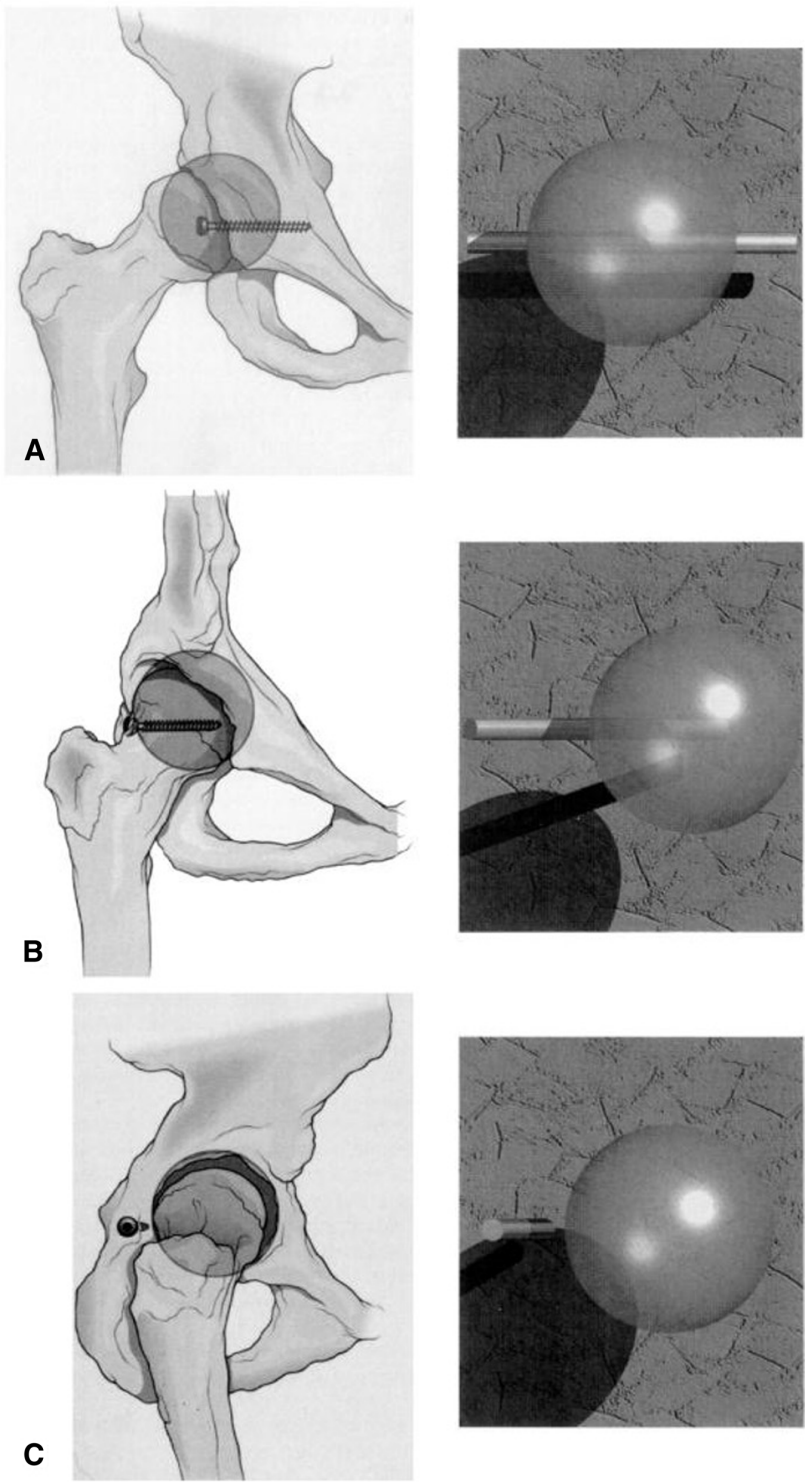
Geometric model of the acetabulum, specifically the posterior wall, demonstrating needing only one view to prove a screw is extraarticular. (**A**) Anteroposterior projection of line (screw) not intersecting sphere. Nonintersection cannot be determined. (**B**) Oblique projection of line (screw) not intersecting sphere. Nonintersection cannot be determined. (**C**) Lateral projection of line not intersecting sphere. As the projection approaches a perpendicular to the line (screw), the relation of the line and the sphere can be determined. [Reprint permission granted 11/11/19, Norris et al. Intraoperative Fluoroscopy to Evaluate Fracture Reduction and Hardware Placement During Acetabular Surgery. J. Orthop Trauma. 1999;13 (6):444–447 [33]

**Fig. 2:**
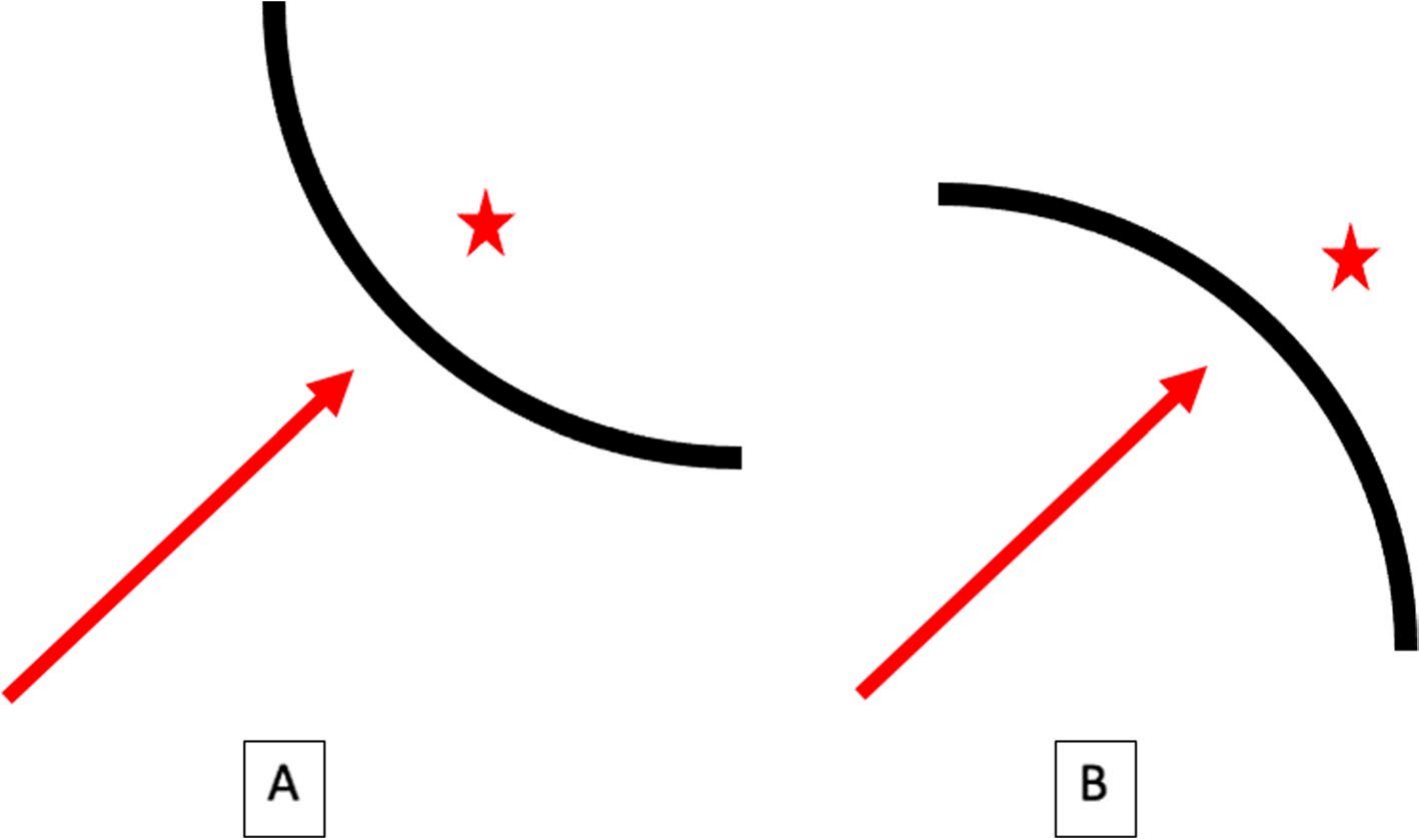
An illustrative diagram summarizing the simplistic approach to placement of periarticular screws. (**A**) When a screw (arrow) is heading towards a concave articular surface (*), only one fluoroscopic view is needed to prove it is beneath the joint; this is exemplified by a screw down the anterior column of the acetabulum. (**B**) When a screw (arrow) is towards a convex articular surface (*), infinite fluoroscopic views must be obtained to prove it is beneath the joint. This is exemplified by a screw within the humeral head

The defining step in placing periarticular screws is to determine whether the articular joint surface the screw is heading towards is convex or concave. If the screw being placed is headed towards a concave articular surface (i.e. the acetabulum’s anterior column), then only one fluoroscopic view is needed to prove it to be extraarticular. This is depicted in Fig. 1, where the screw appears to be within the sphere (the hip joint) when it is in fact outside the confines of it when appropriate views are taken. If the screw is headed towards a convex articular surface (i.e. femoral neck screws heading toward the femoral head), then an infinite number of fluoroscopic views are needed to prove it to be extraarticular. Simple, orthogonal anteroposterior (AP) and lateral views are not sufficient in many cases. This concept can be easily understood when shown in a simple diagram (Fig. 2), allowing extrapolation to all joint and bony surfaces in the body. Several common examples are presented here to illustrate this rationale with regards to the proximal humerus, distal radius, acetabulum and femoral neck, and ankle.

It is worth noting that some may prefer to consider the geometry of the surface the screw is headed towards instead; this can be anthropomorphically described as the screw “having eyes.” If a screw in and across the femoral neck “sees” a convex (albeit non-articular) surface in the femoral head, then the opposite rationale of what we have just described can be applied. We considered this rationale originally but subsequently took a poll at our institution of medical students and residents, and they much preferred to consider the geometry of the surface the cartilage is actually on.

### Proximal Humerus

**Fig. 3:**
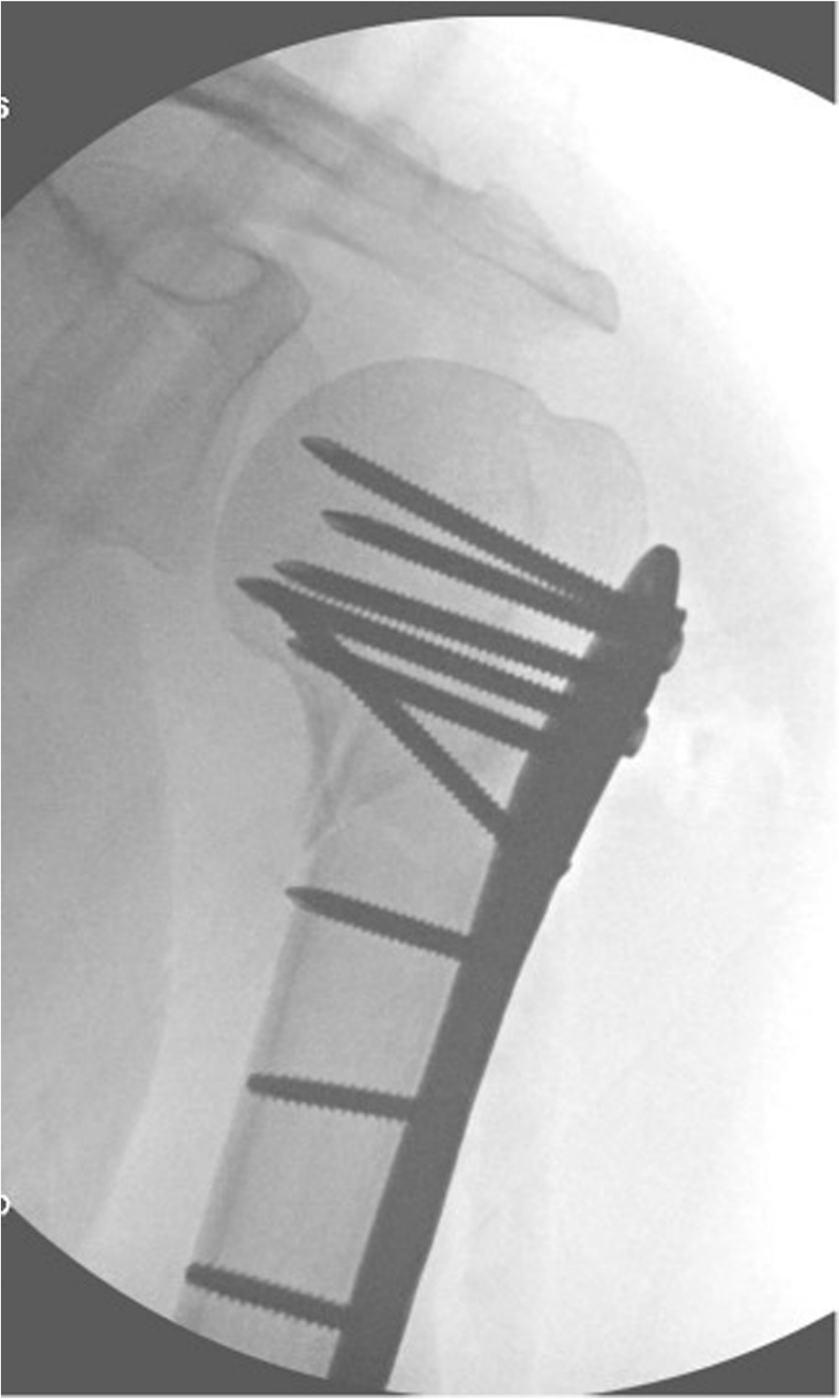
An AP view of a displaced left proximal humerus fracture fixed with a locked plate. The screws in the humeral head are heading towards a convex articular surface, so many more (and technically infinite) views are needed in addition to simple orthogonal views to ensure all the proximal screws are subarticular

Proximal humerus fractures are the second most common fracture of the upper extremity following the distal forearm [7]. Most commonly these are managed nonoperatively, but displacement can warrant arthroplasty or fixation. Many patients with proximal humerus fractures are older with osteoporotic bone, and if plate fixation is pursued it is necessary to maximize stability to prevent postoperative displacement. Locked plating has been shown to improve the biomechanics of fracture fixation in osteoporotic bone [8]. One of the complications of this procedure includes intraarticular placement of the locking screws through the plate’s proximal paddle. Brunner et al. showed that despite the use of fluoroscopy, 22% of patients had intraarticular screw penetration, and 64% of these were at the time of implantation [9]. Due to this common complication and its morbidity, it is imperative to ensure screw placement is extraarticular prior to leaving the operating room. Based upon the rationale described above, screws in the proximal humerus are heading toward a convex articular surface (Fig. 3). Therefore, infinite views are technically needed to ensure that they are not penetrating the humeral head’s cartilage. This means that a screw violating the articular surface on a single view is intraarticular and needs to be shortened or redirected if variable-angle technology is available.

Several techniques have been described to attempt to mitigate the risk of articular screw placement. Screw sounding as described by Bengard et al. made use of the blunt end of a Kirschner wire to reduce the risk of screw perforation through the articular surface [10]. Others have described placing screws shorter than measured; however a potential downside of this technique is the decreased pullout strength of screws as they become farther from the subchondral bone [11]. In a 2015 cadaveric study it was shown that if a screw placed in the proximal humerus remains within the bone, there will be no fluoroscopic image to show that it is intraarticular [12]. However, they showed that an intraarticular screw can appear extraarticular on some views [12]. Advanced imaging techniques have also been described to assess intraarticular screw penetration in the fixation of proximal humerus fractures [13, 14], however these modalities can add additional operative time, radiation to the patient, and prohibit the surgeon from making intraoperative corrections. Jia et al. assessed 134 proximal humerus fractures treated with locked plating and suggested that postoperative CT scans were more reliable than postoperative plain radiographs to visualize screw penetration [13]. However, this study did not examine the accuracy of real-time intraoperative fluoroscopy. Weil et al. sought to compare intraoperative conventional fluoroscopy to a novel three-dimensional (3D) fluoroscopic imaging technique for intraarticular screw visualization in a cadaver model [14]. They found that both fluoroscopic imaging modalities were highly accurate to assess screw penetration in real time [14], which calls into question the need for advanced imaging postoperatively.

At our institution, we track each proximal screw’s length on a Grashey AP view (Fig. 3). At the case’s conclusion, to practically apply “infinite views” we passively rotate the shoulder with live fluoroscopy from full external rotation to full internal rotation (to summate multiple views in between a Grashey and transscapular-Y view) to ensure all the proximal screws are safely within the humeral head. This same technique can be used for the proximal interlocks through an intramedullary nail.

### Distal radius

Distal radius fractures represent one-sixth of all fractures seen in the emergency department and are the most common upper extremity fracture [15]. There has been an ongoing trend towards fixing many distal radius fractures with locked plating. Often with thin, distal articular segments and/or poor bone quality, we try and get our distal screws just barely beneath the articular surface to prevent fixation failure. In recent years, there have been multiple articles published regarding the best fluoroscopic views to obtain in the operating room to decrease the risk of intraarticular and dorsal screw penetration. Based on our rationale presented above, screws placed subchondral through the distal paddle of the plate (whether volar or dorsal) are beneath a concave articular surface below the carpus, so only one view is needed to prove they are not violating the distal radial cartilage. With regards to the tips of screws through a volar plate, this can be problematic and cause extensor tendon irritation and rupture if they breach the dorsal cortex [16]. On their way to the dorsal cortical surface, they are headed towards a convex surface, so infinite views are needed to prove they have not breached.

Soong et al. published an article to determine the best intraoperative fluoroscopic views to avoid articular screw penetration [17]. Authors concluded that due to the curved articular surface of the distal radius, multiple views are needed to rule out intraarticular penetration [17]. To visualize ulnar-sided screws a 15–20 degree lateral tilt is most beneficial, but when visualizing more radial screws one must obtain up to a 30 degree lateral tilt. The dorsal surface of the distal radius is also challenging to evaluate with regard to screw penetration due to the prominence of Lister’s tubercle. Simply getting orthogonal views has fallen out of favor due to the high rate of missed dorsal cortex screw penetration. Recently, the dorsal tangential and 45-degree oblique views have been shown to be the best at detecting dorsal screw penetration [18]. In 2012, Takemoto et al. compared the accuracy of CT scan compared with plain radiographs for evaluating intraarticular penetration of screws in volar locked plating [19]. They concluded that the accuracy was higher for CT scan with higher intraobserver agreement; however, this was not significant. Their limitations included only obtaining a standard set of radiographs that was not necessarily matched to each patient, and there was no ability to conduct live fluoroscopy which has been described as having a very high rate of perforation detection. Work from Wall et al. has shown volar-to-dorsal subchondral screws only need to traverse 75% of the distance to the dorsal cortex for adequate biomechanical stability, so the complication of lengthy screw tips past the dorsal cortex has decreased in most institutions [20].

**Fig. 4:**
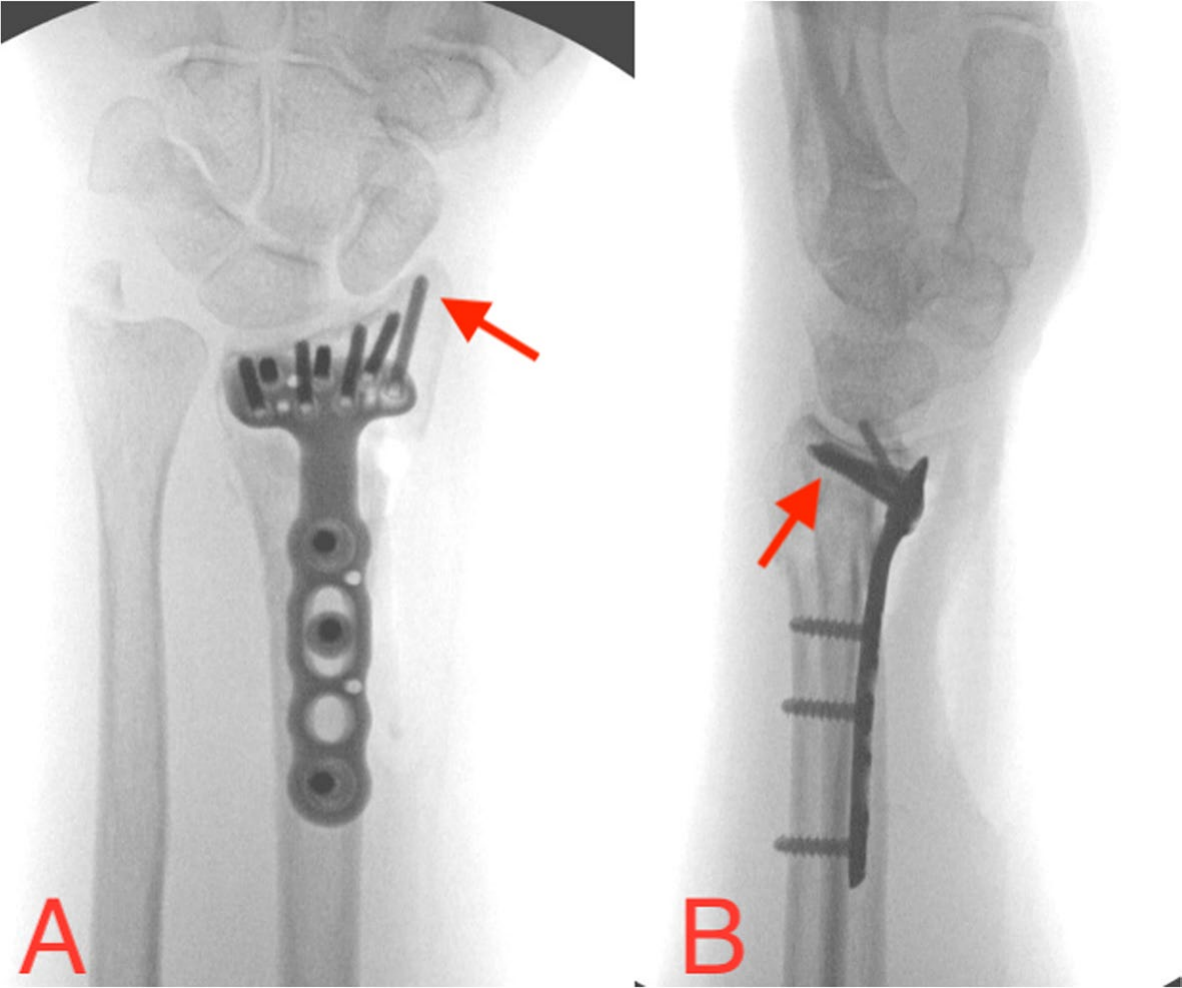
Volar locked plate fixation of a displaced left intraarticular distal radius fracture showing (**A**) a PA view (with volar tilt neutralized) confirming extraarticular placement of the radial styloid screws as they are heading toward a concave articular surface, and (**B**) a lateral view with radial inclination neutralized confirming subchondral non-styloid screws toward a concave articular surface. Screws heading dorsally are headed toward a convex articular surface, so an infinite number of fluoroscopic views are technically needed to judge appropriate screw lengths

At our institution, we simply obtain seven views for all distal radius fracture plating: an AP view, semi-pronated and semi-supinated views, a lateral view with and without radial inclination neutralized (to see the volar lunate and scaphoid facets respectively), a PA (posteroanterior) view with volar tilt neutralized, and a dorsal tangential view. The lateral view with radial inclination neutralized shows the articular surface and can prove the non-radial styloid screws are beneath the articular surface, while the AP or PA can confirm the radial styloid screws are beneath. With regard to the dorsal cortex, when the drill hits or barely goes through the far cortex we take at least 2 mm off for our screw length as described by Park et.al, and the lateral (Fig. 4) and dorsal tangential views (not shown) are used to detect inadvertent penetration [21]. Other, more subtle, techniques are used to make sure the radial styloid screws are neither out the radial cortex (convex surface) nor are the lunate facet screws in the distal radioulnar joint (concave surface). Live fluoroscopy is typically not needed for these cases.

### Acetabulum & Femoral Neck

**Fig. 5:**
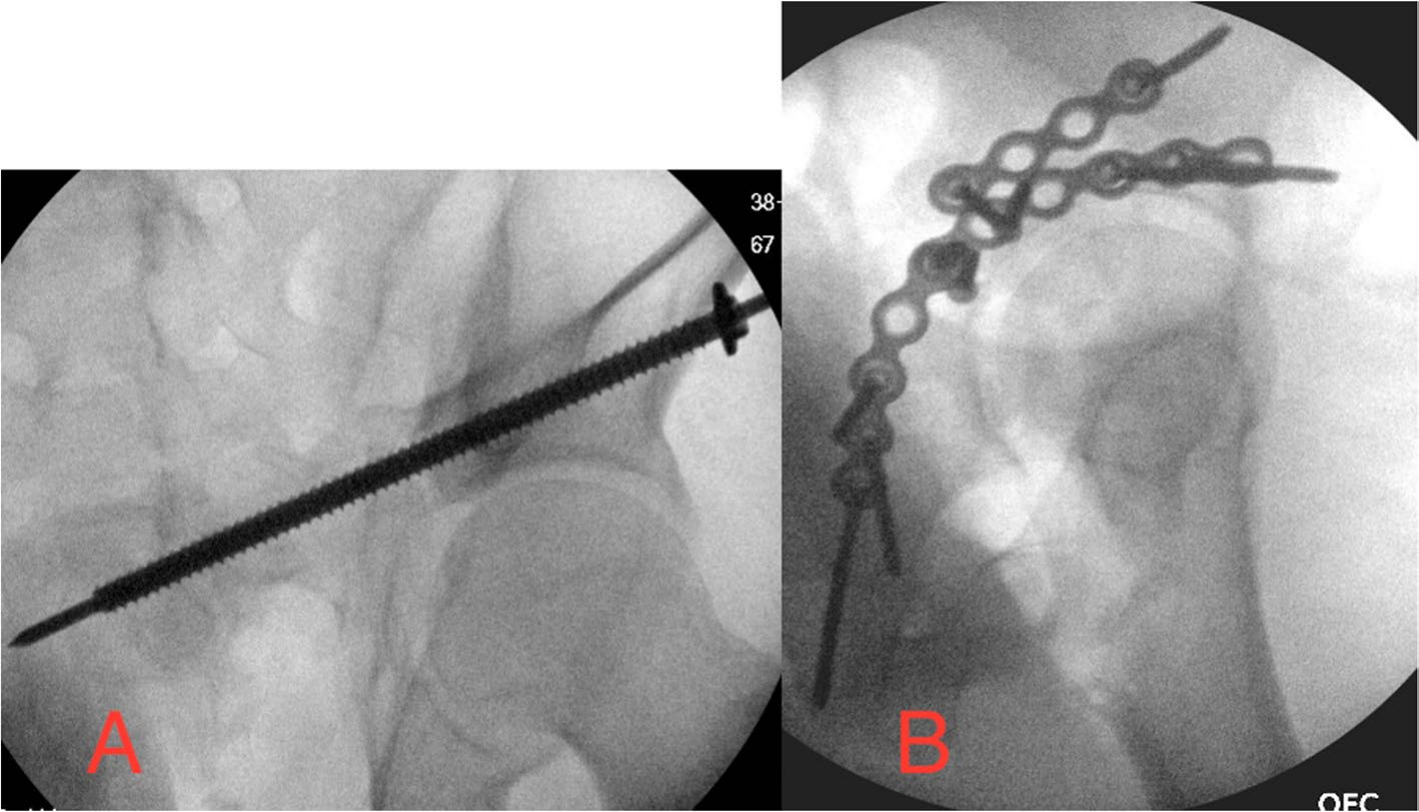
Periarticular screw placement around the acetabulum’s concave articular surface. (**A**) A percutaneous screw across a left anterior column acetabular fracture needs only this one view (obturator-outlet here) to confirm its extraarticular placement. (**B**) Open reduction and internal fixation of a right posterior wall acetabular fracture/dislocation needs only this one lateral view to confirm extraarticular hardware placement

**Fig. 6:**
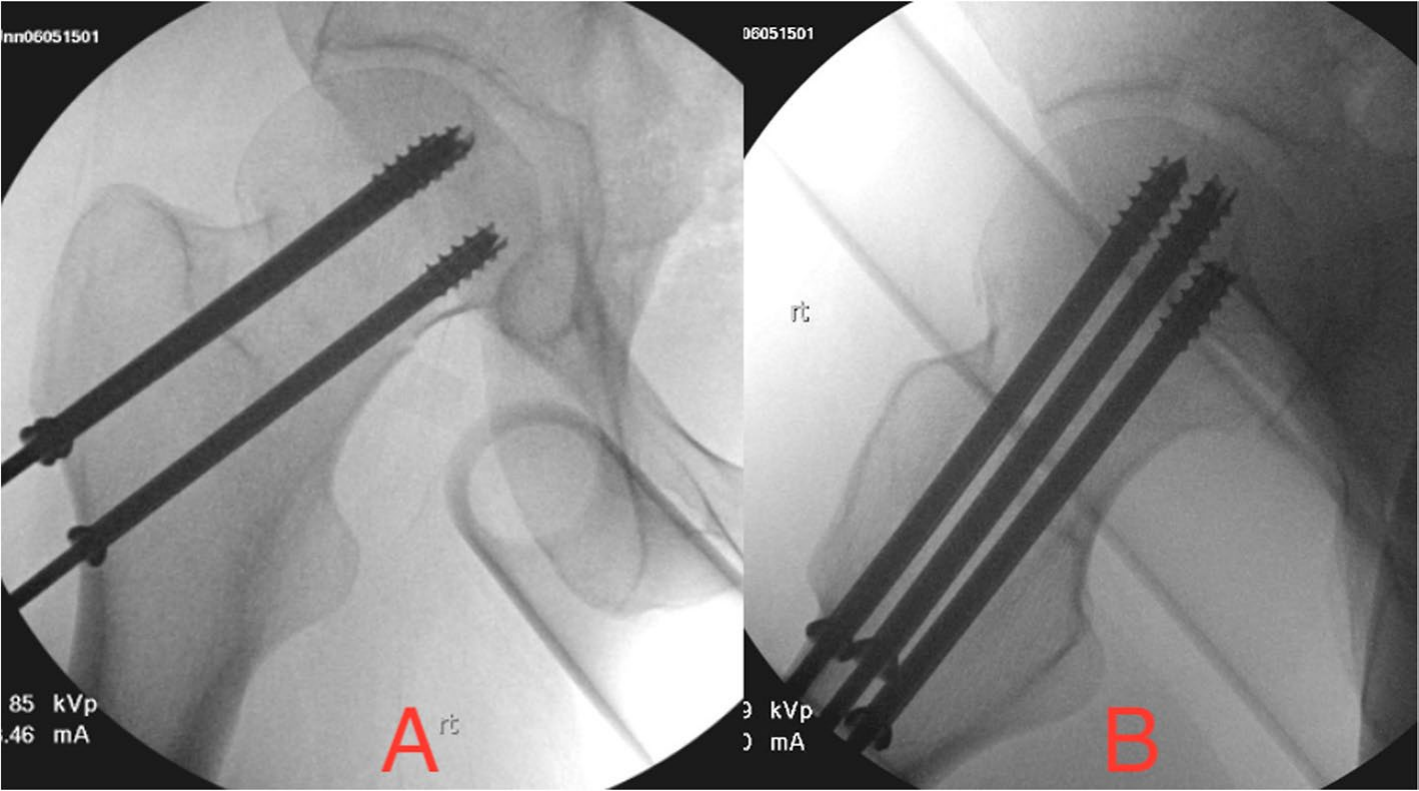
Percutaneous screw fixation of a valgus-impacted right femoral neck fracture. The femoral head is a convex articular surface requiring infinite views to ensure the subchondral placement of the screws. (**A**) shows the AP view, and (**B**) our “instrumentation lateral view”

Surgical fixation of acetabular fractures, femoral neck/intertrochanteric fractures, femoral head fractures, and even slipped capital femoral epiphyses all involve placement of screws close to the hip’s articular cartilage. Periarticular screws at the hip joint involve both sides of the rationale described above. For placement of acetabular screws, because the linear screws are heading towards a concave articular surface, only one fluoroscopic view is needed to prove that a screw is extraarticular. This is represented by an anterior column screw as seen in Fig. 5. When going into the femoral head, the opposite side of our rationale is applied. Screws in the femoral neck in a trajectory toward the head’s articular surface are headed towards a convex articular surface; therefore, infinite views are needed to establish that a screw is extraarticular (Fig. 6).

Substantial literature exists concerning the unintentional placement of intraarticular screws into the hip joint. Because of the spherical shape of the femoral head, simple orthogonal views can fail to detect a point up to 41% of the radius outside a sphere [22]. With the advancement of fluoroscopy, physicians have not had to rely on historical techniques for quite some time. Older modalities have included hip auscultation, initially described in canines, which showed that volunteers were able to correctly determine intraarticular screws [4]. Newer advances in hip arthroscopy and 3D imaging have allowed surgeons to detect intraarticular screws intraoperatively during percutaneous pelvic surgery [5, 23]. Surgeons have also discussed the relevance and appropriate use of postoperative CT scans to evaluate articular penetration of screws in acetabular surgery [24], although this advanced imaging may be unnecessary. Rashidifard et al. recently compared an intraoperative “end on” axial fluoroscopic view to postoperative CT imaging for the positioning of lag screw fixation in 116 posterior wall acetabulum fractures [25]. The “end on” axial view was obtained for each screw using a combination of iliac oblique views with either inlet or outlet tilt. Authors found that screw positioning on intraoperative fluoroscopic “end on” views closely correlated with that of CT imaging, as there was no significant difference in screw localization between the two modalities for screws that were placed < 5 mm from the articular surface. In fact, the postoperative CT measurements were significantly further from the joint than initially visualized on fluoroscopy [25]. As discussed above, Norris et al. evaluated 32 acetabular fractures using meticulous intraoperative fluoroscopy alone to determine extraarticular hardware placement. CT scans of all 32 fixed fractures confirmed extraarticular placement of all the screws [3]. In another study by Archdeacon et al., postoperative CT scans were obtained on 563 of 612 fixed acetabular fracture s[6]. Fourteen patients (2.5%) required revision surgery, and 6 (1.1%) were for inadvertent intraarticular hardware not recognized with intraoperative fluoroscopy and postoperative plain films [6]. Additionally, Carmack et al. called into question the reliability of postoperative CT scans showing the potential for false-positive results from metal artifact leading to unnecessary reoperation [26]. Unfortunately, these techniques demand more operative time, cost, radiation, continued uncertainty, and many do not afford surgeons the ability to make intraoperative corrections to prevent morbidity and reoperation for a screw’s articular penetration. Thus, the ability to accurately place extraarticular hardware using fluoroscopy alone is highly advantageous. By considering the acetabulum as a sphere and the screw as a linear object (Fig. 1), the fluoroscope can be used to try and find an image projection where the acetabulum and screw are separated. When looking through the sphere, one may see the screw to be within the sphere itself when in fact it is not. If separation can be shown between the sphere (acetabulum) and the screw on any single view, then they are not in contact with each other.

With regard to the proximal femur, Hernigou et al. analyzed orthogonal views of the hip after placement of hip screws for femoral neck fractures and found a 25% rate of “technical imperfection”[1]. Of the 60 fractures studied, there were 5 instances (8%) of intraarticular screw penetration and 6 instances (10%) of the screw breaching the femoral neck [1]. This confirmed that simply utilizing AP and lateral X-rays to determine intraarticular penetration of the femoral head (and breach of the femoral neck) is unsatisfactory. It is requisite to prevent perforation of the femoral neck because posterior penetration of the superior neck can disrupt the head’s blood supply and lead to avascular necrosis. Posterosuperior neck penetration as a significant risk was confirmed by Zhang et al., and they recommend redirecting screws that appear against the posterior cortex due to the high probability of perforation [27]. The femoral neck has a very complex nonarticular bony surface with convex and concave undulations. Hernigou et al. described it as a cylinder which makes it most resemble the femoral head when considering our rationale [1]. Recent authors have further proven that an aggressive posterosuperior femoral neck screw that looks safe on fluoroscopy is “in-out-in” the majority of the time with cadaver dissection [28]. The piriformis fossa should now be scrutinized in the coronal plane also to avoid compromise of the femoral head’s critical lateral epiphyseal vessels.

At our institution, when placing a screw across the acetabulum (whether anteriorly or posteriorly), it is headed above a concave articular surface, so only one view is needed to ensure it is outside of the hip joint. Classically for anteriorly-based screws, a Judet projection can prove it out (Fig. 5A). For posteriorly-based screws a lateral projection can prove it out (Fig. 5B). It is often helpful to get the fluoroscope coaxial with any close screw. When placing screws into the femoral head for constructs including percutaneous screws for a valgus-impacted femoral neck fracture, a sliding hip screw for a displaced femoral neck, or lag screws through a cephalomedullary nail for an intertrochanteric fracture, each screw is headed towards a convex surface, Therefore, we need infinite views to ensure they are beneath the hip joint. Practically, this involves an AP (Fig. 6A), a view 10 degrees overrotated, and then views every 20 degrees to a full “anatomic lateral” displaying the neck’s anteversion. Throughout the case, we also obtain an “instrumentation lateral” with the head, neck, greater trochanter, and shaft all in line with the neck’s anteversion neutralized. In a well-done reduction and construct with screws, a sliding hip screw, or a cephalomedullary nail, this shows that the screws are coaxial within coaxial osteology (Fig. 6B).

### Ankle

Fixation of unstable rotational ankle fractures frequently involves screw placement up the medial malleolus, a structure that is an extension of the tibial plafond. These injuries, along with pilon fractures, can be difficult for surgeons to determine if all hardware is extraarticular. In 2011, Giordano et al. showed that surgeons have an extremely low level of agreement when analyzing whether or not medial malleolar screws are intra or extraarticular [29]. They conducted a cadaver study with screws intentionally placed inside of and outside the ankle joint and asked surgeons to evaluate which specimens were thought to be extraarticular based off AP and mortise views. The low level of agreement gave insight into the difficulty of determining acceptable periarticular screw placement and further need for the education this review offers [29]. Others have previously suggested that if using AP and mortise views for placement of medial malleolus hardware, the AP view seems to more accurately predict the location of the screw relative to the subchondral surface [30]. In 2001, Romiti et al. conducted another cadaver study to evaluate medial malleolar screw placement [31]. They concluded that if an X-ray image can show the screw to be extraarticular, then it is in fact extraarticular [31]. Similar results were reported by Wera et al. in 2015 in a study of sawbones where an extraarticular screw could be confirmed with a single fluoroscopic view [32].

**Fig. 7:**
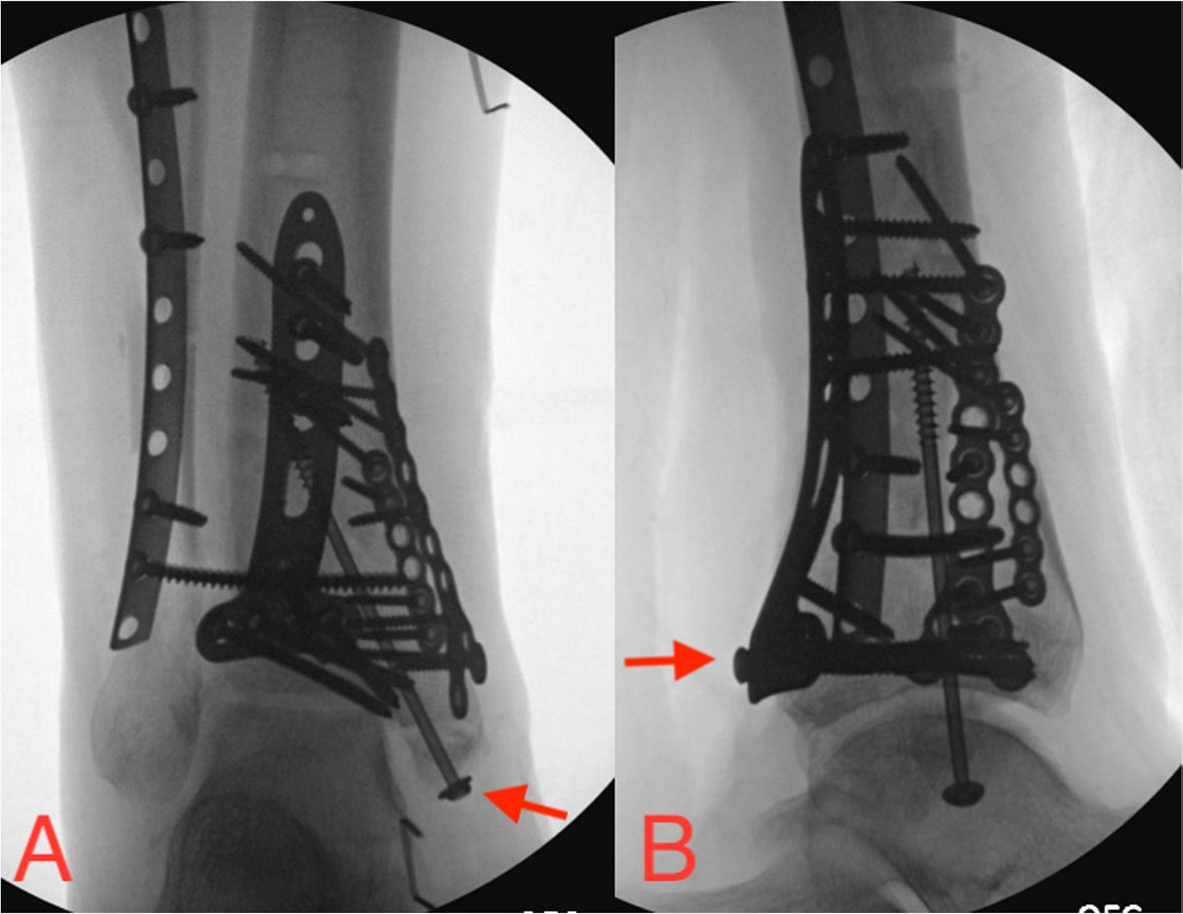
Open reduction and internal fixation of a left pilon fracture. (**A**) A single Mortise view proving the subchondral placement of a medial malleolar screw, as the medial malleolar articular surface is concave. However, this view does not prove that the sagittal-plane plafond screws are extraarticular. (**B**) A single lateral view confirming extraarticular screw placement within the tibial plafond’s concave articular surface

Each of these articles exemplify the rationale presented throughout this paper. At our institution, screws placed up the medial malleolus are heading toward a concave joint surface, which means that if one view can be obtained showing separation of the joint surface and the screw, then it is indeed extraarticular (Fig. 7A). For pilon (and posterior malleolus) fractures it can be difficult for some to realize whether sagittal plane screws are truly above the plafond’s articular surface. These front-to-back or back-to-front screws are traversing above the plafond’s concave surface requiring only one view, typically a perfect lateral (Fig. 7B), to prove that they are outside of the joint.

## Conclusion

Definitively determining whether subchondral screws are safe during the internal fixation of periarticular fractures can be difficult, possibly for lack of dedicated education in residency training and the paucity of review literature. The scientific literature (even recently) in our humble opinion tends to overcomplicate a relatively basic concept. Uncertainty in the operating room can lead to chondral damage and the need for reoperation if screws are unknowingly placed intraarticularly. This uncertainty can manifest in unnecessary tests intraoperatively and/or postoperatively. These adjuncts have historically included auscultation and more recently arthroscopy, CT scanning, and 3D imaging modalities, all of which have downsides in the form of cost, radiation, and/or the inability to make immediate intraoperative adjustments to avoid future surgery that is largely preventable. In our experience, educated use of intraoperative fluoroscopy alone is sufficient to determine safe periarticular screw placement.

This review is not without limitation. While we present several high-yield anatomic locations, not every possible site for periarticular screw placement was discussed. However, understanding the basic principle presented in this review easily allows for extrapolation to every joint surface in the body. Additionally, by nature of a review article, no data was collected nor was a statistical analysis performed. This article simply provides expert opinion backed by an in-depth review of the existing literature. We hope to share these basic principles with all orthopaedic surgeons, specifically those in-training, and create a foundation for their careers. Further research could be considered on this subject to evaluate both the statistical and clinical benefits of using fluoroscopy alone to assess periarticular screw placement.

In summary, if a screw is heading towards a concave articular surface only one view needs to be obtained to prove it is beneath the joint (i.e. anterior column acetabular screw). If a screw is heading towards a convex articular surface, then infinite views must be obtained to prove it is beneath the joint (i.e. femoral neck screws). Infinite views are impossible, so live fluoroscopy with passive rotation of the extremity is used at the proximal humerus, standardized views are used each and every time at the distal radius, and multiple views to a full lateral are used for femoral neck and head screws at our institution. A summary of our intraoperative imaging protocols is depicted in Table 1. We train our residents and fellows that if they command simply what is required at the hip, every other joint in the body we work on can be quickly figured out. By following this simple concept, using fluoroscopy alone can allow all surgeons (specifically residents and non-trauma trained practicing surgeons) to be confident that hardware placement is extraarticular before leaving the operating room. And if a screw is too close to call, change it.

**Table 1:**
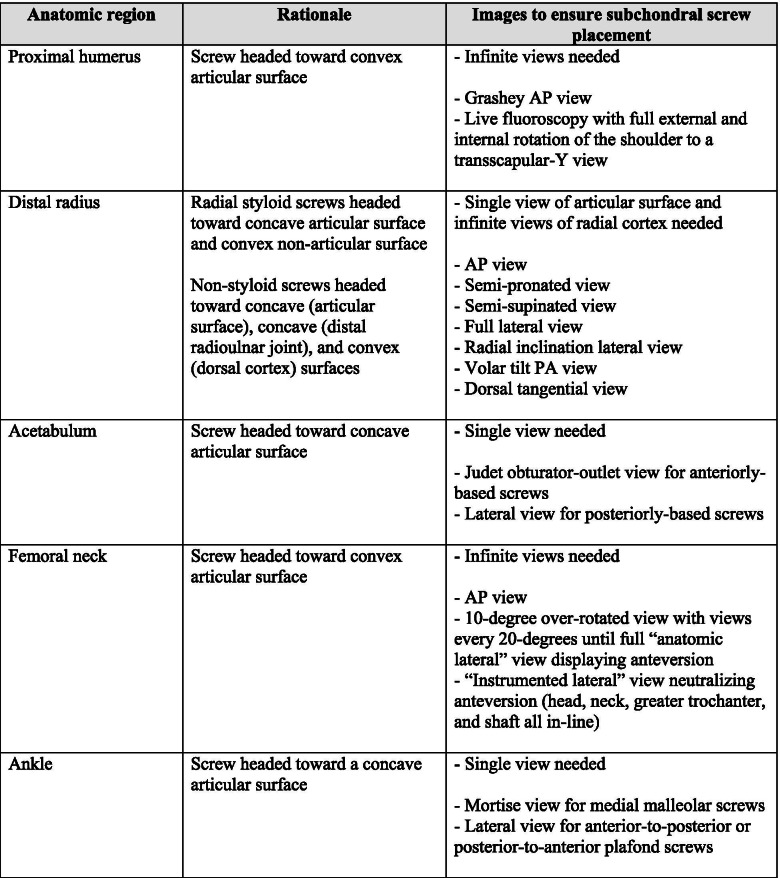
A summary detailing the authors’ intraoperative fluoroscopic imaging protocols to assess periarticular screws and ensure extraarticular placement

## Data Availability

Data sharing is not applicable to this article as no datasets were generated or analyzed during the current study. All sources cited within the manuscript are listed in the references section and available for the reader.

## Abbreviations

CT: Computed Tomography
K-wire: Kirschner wire
3D: Three-dimensional
AP view: Anteroposterior view
PA view: Posteroranterior view
