# Correction to: Periarticular screws: what’s in and what’s out of the ﻿joint?

**DOI:** 10.1186/s12891-022-05255-3

**Published:** 2022-05-17

**Authors:** Michael S. Sridhar, Michael D. Hunter, Michael J. Colello

**Affiliations:** Prisma Health-Upstate Department of Orthopaedic Surgery, University of South Carolina School of Medicine Greenville, 701 Grove Road, 2nd Floor Support Tower, Greenville, SC 29605 USA

**Correction to: BMC Musculoskelet Disord 23, 37 (2022)**

10.1186/s12891-021-04928-9

Following the publication of the original article [1] the authors requested to remove Figure 1 from the article due to unsecured copyright permission to use the figure.

**Fig. 1:**
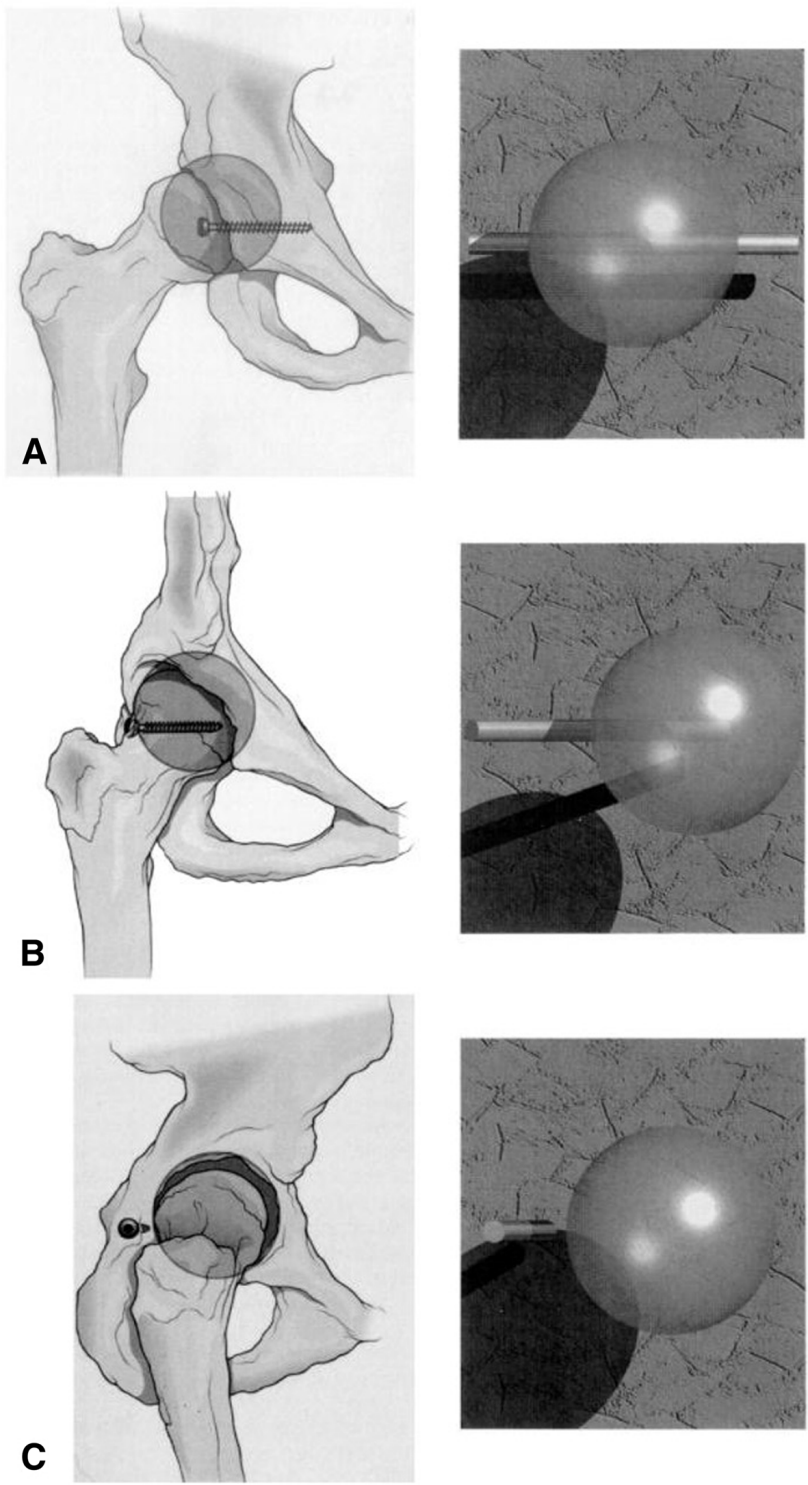
Geometric model of the acetabulum, specifically the posterior wall, demonstrating needing only one view to prove a screw is extraarticular. **A** Anteroposterior projection of line (screw) not intersecting sphere. Nonintersection cannot be determined. **B** Oblique projection of line (screw) not intersecting sphere. Nonintersection cannot be determined. **C** Lateral projection of line not intersecting sphere. As the projection approaches a perpendicular to the line (screw), the relation of the line and the sphere can be determined. [Reprint permission granted 11/11/19, Norris et al. Intraoperative Fluoroscopy to Evaluate Fracture Reduction and Hardware Placement During Acetabular Surgery. J. Orthop Trauma. 1999;13 (6):444–447 [33]

Below is the removed figure.
